# High-Quality Draft Genome Sequence of Biocontrol Strain *Pantoea* sp. OXWO6B1

**DOI:** 10.1128/genomeA.00582-16

**Published:** 2016-06-23

**Authors:** Jennifer Town, Patrice Audy, Susan M. Boyetchko, Tim J. Dumonceaux

**Affiliations:** a Saskatoon Research and Development Centre, Agriculture and Agri-Food Canada, Saskatoon, Saskatchewan, Canada; b Department of Veterinary Microbiology, University of Saskatchewan, Saskatoon, Saskatchewan, Canada; c Québec Research and Development Centre, Agriculture and Agri-Food Canada, Québec, Québec, Canada

## Abstract

*Pantoea* sp. strain OXWO6B1 inhibits the growth of the potato pathogen *Phytophthora infestans*. We determined the 5.2-Mbp genome sequence of this strain, which featured at least 3 confirmed plasmids of up to 250 kbp. The genome sequence of OXWO6B1 is different from that of all previously sequenced strains of *Pantoea*.

## GENOME ANNOUNCEMENT

*Pantoea* sp. strain OXWO6B1 was isolated from seeds of wild oat (*Avena* spp.) near Oxbow, Saskatchewan, Canada. This organism inhibits the growth of the potato pathogen *Phytophthora infestans* on plates and in plant challenge assays and has been identified as a potential biocontrol agent for potato late blight.

*Pantoea* sp. OXWO6B1 was grown at 22°C in a rotary shaker for 24 to 48 h in yeast extract glucose medium (2.0 g/liter yeast extract, 2.5 g/liter glucose, 0.4 mM MgSO_4_·7H_2_O, 0.09 mM MnSO_4_·H_2_O, 0.85 mM NaCl, 0.017 mM FeSO_4_·7H_2_O, 1.84 mM KH_2_PO_4_, and 1.43 mM K_2_HPO_4_). Genomic DNA was purified from 1 ml of overnight culture using the Wizard genomic DNA (gDNA) extraction kit (Promega, Madison, WI, USA). Genomic shotgun sequencing was performed on the MiSeq platform (Illumina), generating 2.9 M paired-end reads. These data were supplemented by an 8-kb-insert paired-end sequencing run using the paired-end rapid library preparation protocol for Titanium chemistry (Roche, March 2012), with modifications as described previously (1). This process generated 187,389 paired-end reads with an estimated pair distance of 6,310 ± 1,577 bp. Illumina reads were assembled using SOAP*denovo*2 (version 2.01), with kmer size of 127 and map length of 34. The resulting 690 contigs (*N*_50_, 44,093 bp) were split into 500-bp pieces with a 200-bp overlap using the EMBOSS splitter, combined with the Roche paired-end reads, and reassembled using Newbler (version 3.0). Gaps in the sequence were filled using the GapCloser tool for SOAP*denovo*2, along with PCR and Sanger sequencing. Assembly of all the sequencing data together produced a high-quality draft (2) genome sequence with 201× coverage featuring 2 scaffolds of 4,675,147 bp (2 scaffold contigs) and 119,254 bp (single contig). Three plasmids of 250 kb, 180 kb, and 4.6 kb were confirmed using PCR. Sequence data were annotated using the Prokaryotic Genome Annotation Pipeline version 3.1 (NCBI) and the Integrated Microbial Genomes portal (https://img.jgi.doe.gov/cgi-bin/mer/main.cgi).

The genome of *Pantoea* sp. OXWO6B1 was 5,234,905 bp (52.74% G+C content). A total of 5,030 genes and 4,868 protein-coding genes were identified, along with 10 genes encoding 5S rRNA, 7 genes encoding 16S rRNA, 7 genes encoding 23S rRNA, and 78 tRNA-coding genes.

The sequences of the taxonomic markers 16S rRNA and *rpoB* (3) suggested that OXWO6B1 was most closely related to *Pantoea ananatis*, with 99% (16S) and >97% (*rpoB*) identity to *P. ananatis*. However, the bacterial barcode marker *cpn60* (4) had a lower sequence identity with any *Pantoea* sp. (the nearest neighbor was *Pantoea stewartii*, with 93.7% sequence identity). Consistent with this, the genomic average nucleotide identity (ANI) of OXWO6B1 was below the specified cutoff for species identity with any reported species of *Pantoea* (5), with a maximum of 88.9% ANI with *P. ananatis*. Moreover, a comparison of 40 Clusters of Orthologous Groups (COGs) using SpecI (6) revealed that *Pantoea* sp. OXWO6B1 could not be assigned to a species cluster (average nucleotide identity was 95.0% to *P. ananatis*). *Pantoea* sp. OXWO6B1 contains genes that have been associated with biocontrol phenotypes, including phenazine carboxylic acid synthesis (7) and cell wall-degradative enzymes (8). Two genes encoding putative beta-lactamases were also observed.

### Nucleotide sequence accession numbers.

This whole-genome shotgun project has been deposited at DDBJ/ENA/GenBank under the accession no. LWLR00000000. The version described in this paper is version LWLR01000000.
